# Genetic Architecture of Resistance to *Alternaria brassicae* in *Arabidopsis thaliana*: QTL Mapping Reveals Two Major Resistance-Conferring Loci

**DOI:** 10.3389/fpls.2017.00260

**Published:** 2017-02-24

**Authors:** Sivasubramanian Rajarammohan, Amarendra Kumar, Vibha Gupta, Deepak Pental, Akshay K. Pradhan, Jagreet Kaur

**Affiliations:** ^1^Department of Genetics, University of Delhi South CampusNew Delhi, India; ^2^Centre for Genetic Manipulation of Crop Plants, University of Delhi South CampusNew Delhi, India

**Keywords:** *Arabidopsis*, *Alternaria brassicae*, disease resistance, necrotroph, QTL mapping, defense response

## Abstract

*Alternaria brassicae*, a necrotrophic fungal pathogen, causes *Alternaria* blight, one of the most important diseases of oleiferous *Brassica* crops. The current study utilized *Arabidopsis* as a model to decipher the genetic architecture of defense against *A. brassicae*. Significant phenotypic variation that was largely genetically determined was observed among *Arabidopsis* accessions in response to pathogen challenge. Three biparental mapping populations were developed from three resistant accessions viz. CIBC-5, Ei-2, and Cvi-0 and two susceptible accessions – Gre-0 and Zdr-1 (commonly crossed to CIBC-5 and Ei-2). A total of six quantitative trait locus (QTLs) governing resistance to *A. brassicae* were identified, five of which were population-specific while one QTL was common between all the three mapping populations. Interestingly, the common QTL had varying phenotypic contributions in different populations, which can be attributed to the genetic background of the parental accessions. The presence of both common and population-specific QTLs indicate that resistance to *A. brassicae* is quantitative, and that different genes may mediate resistance to the pathogen in different accessions. Two of the QTLs had moderate-to-large effects, one of which explained nearly 50% of the variation. The large effect QTLs may therefore contain genes that could play a significant role in conferring resistance even in heterologous hosts.

## Introduction

*Alternaria* spp. are serious necrotrophic plant pathogens, causing a range of diseases in many crops including the *Brassica* species. The most common and destructive diseases of the *Brassica* crops worldwide are caused by four species of *Alternaria* viz. *Alternaria brassicae, Alternaria brassicicola, Alternaria raphani*, and *Alternaria alternata* (Verma and Saharan, 1994). *A. brassicae* is the most invasive on all brassicaceous hosts, though it infects the oil-yielding brassicas preferentially. In the Indian subcontinent, *A. brassicae* is recognized as one of the most important pathogens on oilseed *Brassica* species, especially *Brassica juncea* (Grover and Pental, 2003). *Alternaria* blight causes a reduction in photosynthetic potential; results in abnormal seed development, and a reduction in seed oil content and quality leading to 10–71% yield losses worldwide (Saharan et al., 2015). Extensive screening of *B. juncea* germplasm has shown a lack of complete resistance to *A. brassicae*.

*Arabidopsis thaliana* has been used as a model host to study the pathogenesis of various plant pathogens. The extensive natural variation and the vast array of genomic and molecular resources available in *Arabidopsis* make it a genetically tractable system for unraveling the molecular components of host defense. Numerous reports of inter-accession variation in host responses to pathogens have been documented (Hinsch and Staskawicz, 1996; Deslandes et al., 1998; Denby et al., 2004; Lorang et al., 2004; Aranzana et al., 2005; Nemri et al., 2010).

Studies on *A. brassicicola* in the model species *Arabidopsis thaliana* have largely been restricted to studying the transcriptomic and proteomic responses of the host to the fungal invasion (Thomma et al., 1999; Narusaka et al., 2003; van Wees et al., 2003; Mukherjee et al., 2010). Though these studies have provided valuable information regarding the involvement of different pathways in resistance to the pathogen, the genetic basis of resistance has not been elucidated. Kagan and Hammerschmidt (2002) described qualitative phenotypic variation of *Arabidopsis* accessions in response to *A. brassicicola*, but the genetic basis of such variation was not investigated.

Given the importance of resistance to *A. brassicae* in *Brassica* species where no resistance is known and the lack of studies defining the genetics of resistance, a comprehensive study of the genetic determinants of resistance to *A. brassicae* is warranted. Here, we aim to decipher the genetic architecture of resistance to *A. brassicae* in *Arabidopsis*. A phenotypic screen revealed contrasting responses to pathogen challenge with high heritability suggesting that substantial amount of phenotypic variation can be explained by genetic factors. To determine whether multiple genetic factors or a few shared large-effect factors impart resistance in different genetic backgrounds we carried out QTL analysis in three different biparental mapping populations. These populations were developed by crossing three resistant accessions with two susceptible accessions. Our data reveals that two major QTLs which explain a significant amount of phenotypic variation and several minor QTLs are involved in resistance to *A. brassicae*.

## Materials and Methods

### Plant Lines

Seeds of the 15 *Arabidopsis thaliana* accessions were obtained from *Arabidopsis* Biological Resource Center (ABRC) (Supplementary Table S1). Seeds were surface sterilized, cold stratified for 3 days and were grown on MS medium supplemented with 1.5% agar. Seedlings were then transferred to six-inch pots containing Soilrite mix (Soilrite: Perlite: Vermiculite mixed in the ratio of 4:1:1) pre-soaked with nutrient solution. The plants were grown at 22°C and 60% humidity, under 10-h/14-h light/dark cycle for 5 weeks before challenging them with the pathogen.

### *Alternaria brassicae* Culture Conditions and Pathogenicity Assays

The isolate of *Alternaria brassicae* (J3) utilized in the current study was collected from the experimental field station in the village of Jaunty (Delhi), purified, and maintained as single spore cultures for further use. For *in vitro* culture and maintenance of *A. brassicae* isolates, radish root sucrose agar (RRSA) medium was used (Thakur and Kolte, 1985). Fungal cultures were subcultured every fortnight and maintained at 22°C under 12-h/12-h light/dark cycle. To maintain the virulence of the strain, the pathogen was periodically given a passage through *B. juncea* var. Varuna (its natural host) and the reisolated fungus used for pathogenicity assays.

Spores from 15-day old cultures were used for infections. Cultures were flooded with distilled water to collect spores, and the resulting spore suspension was filtered through two layers of muslin cloth. The filtered spore suspension was adjusted to a spore concentration of 3–5 × 10^3^ spores mL^-1^ before inoculation. Inoculations were done on 5-week old *Arabidopsis* plants by drop inoculation method (Xu and Ko, 1998). A total of 8–10 similarly aged leaves per plant were marked for infection. Four droplets of 5 μL spore suspension were applied onto the adaxial surface of the true leaves. Inoculated plants were kept at 100% humidity at 20 ± 1°C under a 10-h/14-h light/dark cycle. Infected plants were evaluated for symptoms 7 days post infection (dpi).

### Disease Evaluation

The disease was discerned by the formation of necrotic lesions that were encircled by chlorotic halos. Different evaluation criteria were used for the RIL and F_2_ populations. The RIL population was evaluated using a DI score where the number of necrotic spots developed was taken into account (McKinney, 1923). Since the F_2_ populations were scored on the basis of the disease reaction from single F_2_ individuals, the plants were scored not only for the number of necrotic spots developed but also the size of the spot. The cumulative score was thus used to model the disease spread more finely. Additionally, the cumulative DI (CDI) data was normalized to the susceptible parent.

The RIL population was quantitatively scored on the basis of DI calculated from the number of necrotic spots using the formula:

$$\begin{array}{c} DI=\frac{\sum_{n=1}^{K}\left(\mathrm{ns}\right)}{K\times {\mathrm{ns}}_{\max}} \end{array}$$

where,

DI = Disease Index

K = Total no. of leaves (usually 8)

ns = No. of necrotic spots/leaf

ns_max_ = Maximum no. of necrotic spots (usually 4).

The F_2_ populations were scored on the basis of CDI calculated from the number of necrotic spots and the size of each spot using the formula:

$$\begin{array}{c} CDI=\frac{\sum_{n=1}^{K}\left(\mathrm{ns}\times \mathrm{ss}\right)}{K\times {\mathrm{ns}}_{\max}\times {\mathrm{ss}}_{\max}} \end{array}$$

where,

CDI = Cumulative Disease Index

K = total no. of leaves

ns = total no. of necrotic spots/leaf

ss = size of the necrotic spot

ns_max_ = maximum no. of necrotic spots (usually 4)

ss_max_ = maximum size of the necrotic spot.

### Genomic DNA Isolation

DNA isolation from *Arabidopsis* leaves was carried out using a rapid extraction protocol (Edwards et al., 1991). Briefly, leaves (<100 mg) were crushed in 500 μL of Extraction buffer [200 mM Tris-HCl, 250 mM NaCl, 25 mM EDTA (pH 8.0), 0.5% SDS] and centrifuged at 13,000 rpm for 5 min to remove the debris. An equal amount of isopropanol was added to the supernatant to precipitate the DNA and centrifuged again at 13,000 rpm for 10 min. The pellet was washed with 70% ethanol, air-dried, and resuspended in 50 μL of 2 mM Tris (pH 8.0). Genomic DNA extraction for SNP genotyping was carried out using MDI (Advanced Microdevices, Pvt. Ltd, India) plant genomic DNA miniprep kit according to the manufacturer’s instructions.

### Development of Mapping Populations and Construction of Genetic Maps

The Cvi-0 (♀) × Gre-0 (♂) RIL (CvG) population, consisting of 125 individuals, was generated by single-seed descent method from a single F_1_ hybrid. The population was advanced up to F_7_ generation before use.

Two F_2_ populations were generated using CIBC-5 and Ei-2 as the female parent and Zdr-1 as the common male parent. Mature single siliques containing F_1_ seeds were harvested separately, and seeds from a single silique per cross were grown. DNA from each plant was isolated, and heterozygosity of the F_1_ plants was assessed using five polymorphic markers from across the genome. The F_1_ plants were allowed to self-pollinate, and the F_2_ seeds were collected and used for phenotyping. A total of 220 F_2_ individuals from the CIBC-5 × Zdr-1 (CZ) cross and 180 individuals from the Ei-2 × Zdr-1 (EZ) cross were phenotyped.

A linkage map for the CvG population was constructed using the genotypic data of 125 RILs for 202 markers (75 SSR, 86 AFLP, and 41 IP markers) using the JoinMap program (Version 4.1) (Supplementary Figure S1; Supplementary Table S2). SNP markers were used for constructing the linkage maps in the CZ and EZ populations. Polymorphic SNPs between the two parents were selected using the Multiple SNP Query Tool (MSQT) (Warthmann et al., 2007). SNP genotyping was carried out using KBioscience KASPar assay. The primer design, assay development, and genotyping were undertaken by KBiosciences Pvt. Ltd (Hoddesdon, UK). A total of 152 SNP markers (evenly distributed across the genome) were used for genotyping each of the F_2_ populations and linkage maps for both the populations were constructed using the JoinMap program (Version 4.1) (Supplementary Figures S2 and S3; Supplementary Tables S3 and S4).

### QTL Analysis

The DI and normalized CDI (NCDI) were used as the trait values for QTL mapping in the RIL and F_2_ populations, respectively. QTLs were detected with QTL Cartographer 2.5 by composite interval mapping (Wang et al., 2012). Genome scan for QTLs was carried out at a walk speed of 2 cM. Statistical significance of QTL for each trait was assessed by performing 1000 permutation tests with a significance level of 0.05 (Churchill and Doerge, 1994; Doerge and Churchill, 1996). A minimum LOD value of 2.5 for the RIL and 3.1 for the F_2_ populations was used for the identification of putative QTLs based on the permutations tests. The additive effects and contribution of each QTL to phenotypic variance (R^2^) were estimated from the composite interval mapping results. QTLs were named as **R**esistance **t**o ***A****lternaria* ***b****rassica****e*** [population name] [chromosome number-1].

## Results

### Screening of *Arabidopsis* Accessions in Response to *Alternaria brassicae*

**FIGURE 1:**
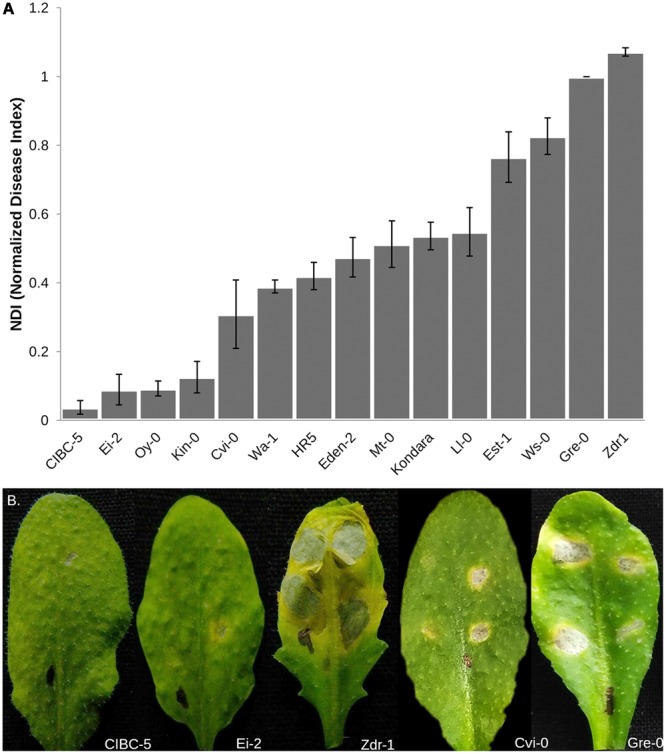
**Phenotypic variation in response to *A. brassicae* in 15 different accessions of *Arabidopsis*. (A)** Normalized Disease Index (NDI) scores of different *Arabidopsis* accessions calculated 7 dpi. The data shown represents the mean and standard error (SE) of atleast 10 plants over three independent experiments. Accessions are shown in increasing order of NDI. **(B)** Representative leaves of *Arabidopsis* accessions used to generate the mapping populations, showing contrasting responses to *A. brassicae* infection. Susceptible accessions Zdr-1 and Gre-0 show typical spreading necrotic lesions 7 dpi, whereas pinhead lesions were observed in the resistant accessions.

Phenotypic variation in resistance to *A. brassicae* was tested in 15 accessions of *Arabidopsis* belonging to different geographical origins (Supplementary Table S1). The accessions showed considerable variation in their response to *A. brassicae* (**Figure 1A**). The accessions displayed variation in the frequency of lesion formation (presence/absence of necrotic lesions) as well as the size of the necrotic lesions formed in response to pathogen challenge. The resistant accessions displayed pinhead-sized necrotic lesions at very low frequencies whereas the highly susceptible accessions had lesions expanding beyond the droplet size at almost all the inoculated spots. The expanding lesions in the highly susceptible accessions occasionally coalesced to form a single large lesion. Typical chlorotic halos encircling the necrotic lesions were also observed in the susceptible accessions. The normalized DI (NDI) derived from the incidence of lesion formation, varied from 0.03 (CIBC-5) to 1.07 (Zdr-1). The variation in NDI was largely genetically determined as indicated by the broad sense heritability score (H^2^ = 0.90). Three different mapping populations were developed from the highly resistant and susceptible accessions to dissect the genetic basis of resistance to *A. brassicae*.

### Phenotypic Variation of the Mapping Populations

**FIGURE 2:**
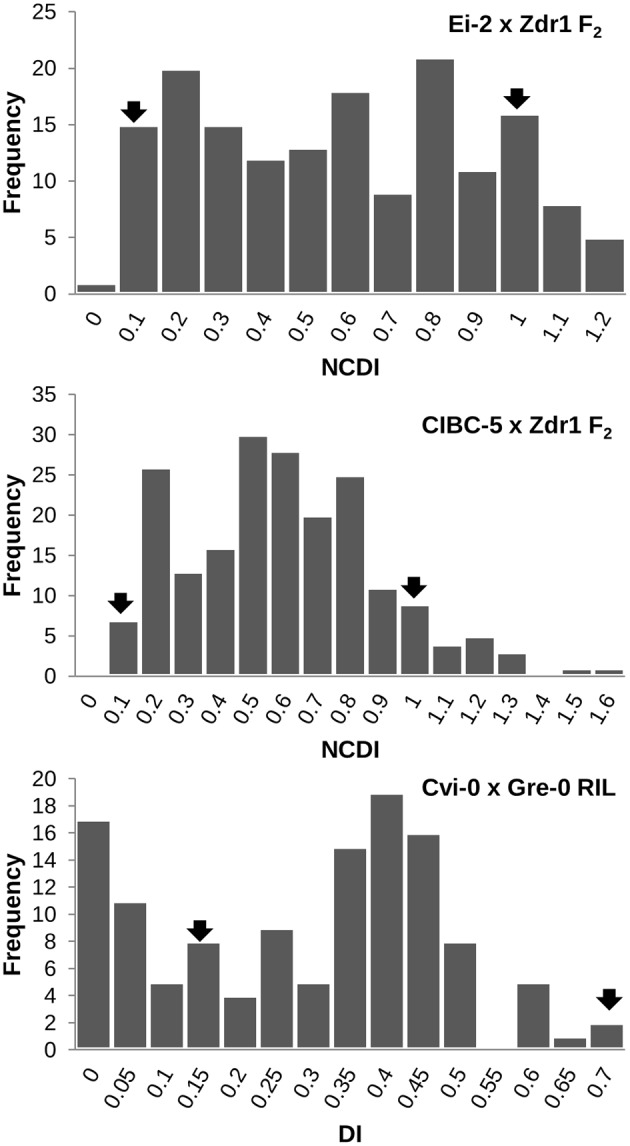
**Frequency distribution showing the range of DI/NCDI in the three mapping populations (EZ, CZ, and CvG).** The mean of the parental accessions are indicated by arrows in the figure. Transgressive segregants can be seen in all the three populations.

The contrasting disease reactions of the parental accessions are given in **Table 1** (**Figure 1B**). The phenotype of the F_1_ progeny from each of the three crosses was intermediate to that of the respective parental accessions, however, they were closer to the resistant parent than the susceptible one (**Table 1**). The DI in the CvG population ranged from 0 to 0.671 of which 17 lines showed no infection. The frequency distribution based on the DI showed nearly a bimodal distribution (**Figure 2**). The CDI in the CZ population varied continuously and was normally distributed with a positive skew while the CDI in the EZ population had nearly a bimodal distribution (**Figure 2**). Few transgressive segregants were observed in all the mapping populations. Heritability in the three populations showed that a significant proportion of the phenotypic variation (∼50%) could be explained by genetic factors (**Table 1**).

**Table 1:** Summary of the phenotype (DI and CDI) of parental accessions, F_1_ progeny, and the mapping populations.

	Mean ± SE	Range	Heritability^a^ (H^2^)
**Cvi-0** × **Gre-0 population**			
Cvi-0	0.2437 ± 0.0783	0.156–0.400	
Gre-0	0.8062 ± 0.0189	0.500–1.000	
F_1_	0.2717 ± 0.0472	0.175–0.452	
RIL	0.2651 ± 0.0165	0.000–0.671	0.4939
**CIBC-5** × **Zdr-1 population**			
CIBC-5	0.0908 ± 0.0111	0.000–0.234	
Zdr-1	0.6219 ± 0.0192	0.422–1.000	
F_1_	0.3583 ± 0.0461	0.167–0.583	
F_2_	0.3238 ± 0.0126	0.025–0.999	0.5614
**Ei-2** × **Zdr-1 population**			
Ei-2	0.1189 ± 0.0139	0.004–0.313	
Zdr-1	0.6219 ± 0.0192	0.422–1.000	
F_1_	0.3987 ± 0.0374	0.083–0.708	
F_2_	0.3326 ± 0.0161	0.000–0.747	0.5892

*^a^Heritability (H^2^) indicates the amount of variation in the population that is genetically determined.*

### QTL Mapping for Disease Resistance

In the CvG population, four QTLs were identified. The major QTL RtAbe_CvG_2-1 was located on the long arm of chromosome 2 at around 35.5 cM in the CvG genetic linkage map (**Table 2**). The confidence interval based on a 2 LOD score drop from the peak placed RtAbe_CvG_2-1 between 27.9 and 48.1 cM covering 12 markers. The major QTL explained up to 42% of the phenotypic variance. Other minor QTLs cumulatively explained up to 19% of the variance. The Gre alleles of RtAbe_CvG_2-1 and RtAbe_CvG_1-1 increased the DI while those at RtAbe_CvG_4-1 and RtAbe_CvG_3-1 decreased the DI.

**Table 2:** Quantitative trait locus (QTLs) governing resistance to *A. brassicae* in the three *Arabidopsis* mapping populations.

QTL	Chr	LOD^a^	Peak LOD position (cM)	Physical intervals (genes flanking the QTL)	QTL effects^b^		Phenotypic variance explained (R2)^c^
					Dominance	Additive	
**Cvi-0** × **Gre-0 population**							
RtAbe_CvG_2-1	2	22.61	35.5	At2g18950–At2g34100	-	-0.1511	0.419
RtAbe_CvG_4-1	4	6.04	52.8	At4g21445–At4g40100	-	0.0608	0.084
RtAbe_CvG_1-1	1	4.81	26.82	At1g06320–At1g11370	-	**-**0.0558	0.062
RtAbe_CvG_3-1	3	3.61	15.71	At3g09540–At3g16760	-	0.0466	0.045
**CIBC-5** × **Zdr-1 population**							
RtAbe_CZ_5-1	5	8.01	57.9	At5g42970–At5g48400	-0.0499	-0.1786	0.113
RtAbe_CZ_3-1	3	4.1	68.7	At3g49280–At3g52480	**-**0.0045	0.1183	0.079
RtAbe_CZ_2-1	2	3.94	36.6	At2g24090–At2g26880	**-**0.0134	**-**0.1745	0.065
**Ei-2** × **Zdr-1 population**							
RtAbe_EZ_2-1	2	20.93	37.6	At2g19900–At2g28950	0.114	-0.2931	0.566

*^a^Logarithm of odds(LOD) of the identified QTL. ^b^Dominance and additive effect of the QTL on the disease index. ^c^The phenotypic variance accounted for by each QTL.*

In the CZ population, three QTLs were detected. The major QTL RtAbe_CZ_5-1 located on the long arm of chromosome 5 was positioned around 57.9 cM in CZ linkage map (**Table 2**). The RtAbe_CZ_5-1 QTL spanned four markers between 53.4 and 63.3 cM. RtAbe_CZ_5-1 explained approximately 11% of the phenotypic variance while RtAbe_CZ_3-1 and RtAbe_CZ_2-1 explained approximately 8% and 6% of the phenotypic variance respectively. The Zdr-1 allele at RtAbe_CZ_2-1 and RtAbe_CZ_5-1 increased the CDI whereas the Zdr-1 allele at RtAb_CZ_3-1 decreased it. The presence of QTLs with opposing additive effects (RtAb_CZ_3-1 vs. RtAbe_CZ_2-1 and RtAbe_CZ_5-1) may thus be responsible for the transgressive segregants observed in the population.

In the EZ population, a single major QTL was detected. The QTL RtAbe_EZ_2-1 located on chromosome 2 with the maximum LOD score positioned around 37.6 cM, covered four markers between 36.6 and 39.7 cM (**Table 2**). The QTL explained nearly 57% of the phenotypic variance. The Zdr-1 allele at this QTL increased the CDI. This locus also displayed a moderate dominance effect (*D* = 0.1140), wherein the Ei-2 allele was partially dominant over the Zdr-1 allele.

**FIGURE 3:**
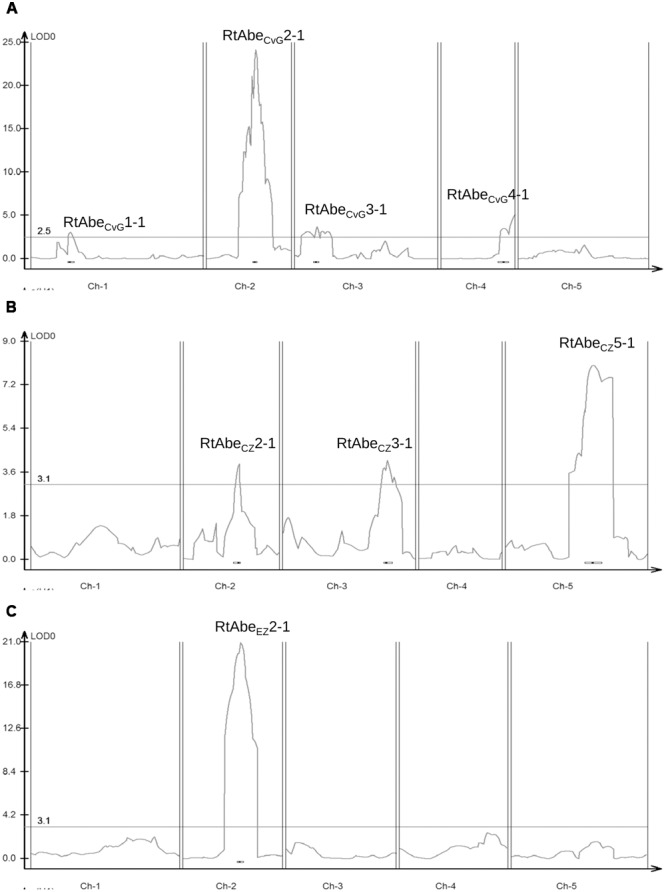
**Quantitative trait locus (QTL) likelihood map of the three mapping populations – (A)** CvG RIL population; **(B)** CZ F_2_ population; **(C)** EZ F_2_ population. Horizontal line denotes the threshold LOD value for the respective populations. The QTLs on chromosome 2 viz. RtAbe_CvG_2-1, RtAbe_CZ_2-1, and RtAbe_EZ_2-1 can be seen to align approximately in the same physical interval.

A total of eight QTLs were identified in the three populations out of which three were major QTLs (**Table 2**; **Figure 3**). Since, the physical positions of most of the markers (except AFLP markers) are known, comparisons can be made across the populations even though different set of markers have been used for different populations. The QTLs identified on chromosome 2 in the three populations had overlapping physical intervals and thus could be said to be a common QTL though it had varying effect sizes in different populations. Thus, six QTLs were identified in the study, of which one is a common QTL and other five are population-specific.

### Putative Candidate Genes within the QTL Intervals of the Major Loci

Since the markers used in the construction of the genetic maps correspond to physical positions in the *Arabidopsis* genome, all the annotated genes in the QTL intervals could be easily identified. The RtAbe_CvG_2-1 QTL from the CvG population was flanked by the markers in the genes At2g18950 and At2g34100. This 6.2 Mb interval contained 1609 protein-coding genes. The RtAbe_CZ_5-1 QTL from the CZ population was flanked by markers in the genes At5g42970 and At5g48400. A total of 601 protein-coding genes were found in this 1.8 Mb interval. The markers in the genes At2g19900 and At2g28950 flank the RtAbe_EZ_2-1 QTL from the EZ population, and this 1.6 Mb region contained 958 protein-coding genes. Also, the minor QTL RtAbe_CZ_2-1 from the CZ population approximately lies in the same physical interval flanked by the genes At2g24090 and At2g26880.

Subsequently, these QTL intervals were examined for genes with known functions in disease resistance. A GO term enrichment analysis of the genes in each QTL interval was carried out. A list of genes with the GO term – response to stress and other related GO terms was compiled. The RtAbe_CvG_2-1 QTL consisted of 55 probable candidate genes that were annotated with known functions in disease resistance pathways. The RtAbe_EZ_2-1 QTL consisted of 33 such genes, which overlapped with the genes in the RtAbe_CvG_2-1 QTL. Some of the possible candidate genes/gene families with a role in host defense in this region include *WRKYs, DEFLs* (Defensin-like), *EIN3*, Glycosyl hydrolases, *PR* (pathogenesis-related) genes, lipid acyl hydrolase and *ERFs* (ethylene response factors).

**FIGURE 4:**
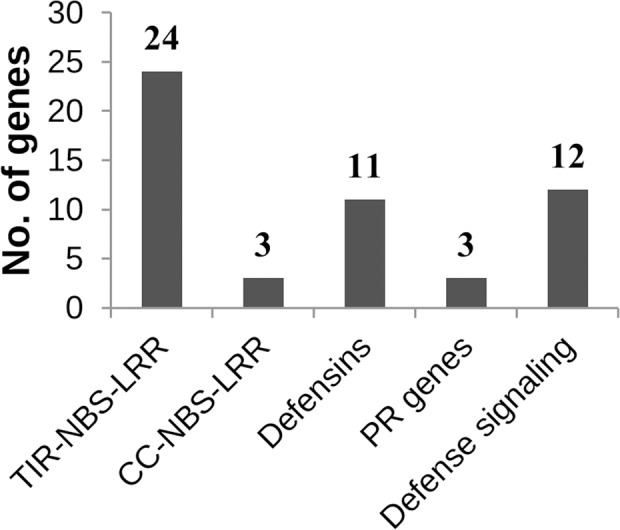
**Classification of known defense response genes in the RtAbe_CZ_5-1 QTL interval.** The TIR- and CC-NBS-LRR genes involved in recognition represent the first layer of the defense cascade while the defensins and the PR genes represent the end product of the defense signaling cascade.

The RtAbe_CZ_5-1 QTL on chromosome 5 was significantly enriched for GO terms such as defense response and response to stress (Supplementary Table S5). A total of 27 disease resistance genes were located in this region out of which 24 were TIR-NBS-LRR class, and three were CC-NBS-LRR class. Other possible candidate genes in this region that are involved in defense signaling pathways include *BIR1* (BAK1-interacting receptor-like kinase), *MYC3* (JAZ-interacting transcription factor), *ERFs* (Ethylene response factors), *BRG* (Botrytis-induced Related Gene), *RBOHD* (Respiratory Burst Oxidative Homolog D) and *NPR3* (Non-expressor of PR3). Additionally, this interval contained nearly 14 defensin and putative defensin-like genes that are known to have antimicrobial /antifungal activity against many pathogenic fungi (**Figure 4**).

## Discussion

The work reported here explores the genetics of resistance to the necrotrophic pathogen *A. brassicae* in *Arabidopsis* by using three different biparental populations to map the underlying components imparting resistance. The continuous variation exhibited by the *Arabidopsis* accessions and the high heritability (H^2^) points to the fact that a large part of the variation is contributed by genetic factors. The mapping populations developed from the contrasting parental accessions also showed a continuous distribution in the disease indices. The F_1_ progeny of the three mapping populations displayed an intermediate phenotype that is consistent with a quantitative nature of the trait with mostly additive effects. Interestingly, in the EZ population, the QTL identified had a moderate dominance effect with the allele from the resistant parent being dominant over the allele from the susceptible parent. The transgressive segregants observed in the mapping populations may represent events where a combination of alleles from both parents increased the trait value beyond the parental values.

A comparative analysis of the QTLs from the three populations revealed that RtAbe_CvG_2-1, RtAbe_CZ_2-1, and RtAbe_EZ_2-1 from the three populations were present in approximately the same physical interval (**Table 2**; **Figure 3**). The F_2_ populations, CZ and EZ share a common susceptible parent; therefore any variation in the same loci (RtAbe_CZ_2-1 and RtAbe_EZ_2-1) would result from allelic variation among the resistant parents viz. CIBC-5 and Ei-2. The overlap of RtAbe_CvG_2-1 in the same physical interval may indicate that there could be a different resistance allele in the same gene or a different gene in the same physical interval, which confers resistance. Moreover, the common QTL is a major resistance conferring locus in the CvG and EZ populations while in the CZ population, it is a minor QTL. This suggests that, if it is the same gene in all the three mapping populations, then the effects of the alleles are conditional on the genetic background. A total of five population-specific QTLs were identified in the current study (**Figure 3**). Since population-specific QTLs of small effects were detected in two of the mapping populations, it does not seem that population size or marker density contributed to the mapping of different QTLs in different populations. Also, since the mapping populations were grown and infected under controlled conditions, the environmental variation can be considered to be minimal. Therefore, these analyses suggest that the population-specific QTLs detected are due to genetic variation that is partitioned among the natural accessions of *Arabidopsis*. This is additionally supported by the high broad sense heritability estimate of resistance among the natural accessions. The presence of both common and population-specific QTLs points to the fact that resistance to *A. brassicae* is multigenic, and that different genes/mechanisms might be activated in various accessions in response to the same pathogen. Unlike in the case of other necrotrophs such as *Botrytis cinerea* where multiple loci of small-to-medium effects were identified (Denby et al., 2004), we have identified two major loci, having medium-to-large effects conferring resistance to *A. brassicae*.

The polygenic or quantitative nature of host defense is due to the additive effect of multiple genes required to mount an effective resistance response against the various virulence strategies of a necrotroph. In addition to the additive effects, gene–gene interactions and gene–environment interactions also contribute to the quantitative nature of the trait. The additive component of the genetic variation is the most commonly encountered variation in most quantitative traits. In the current study also, the QTLs RtAbe_CZ_5-1 and RtAbe_CZ_2-1 are additive in nature, and the presence of both these QTLs may confer durable resistance. This is supported by the fact that CIBC-5, which contains the resistance alleles at the QTLs RtAbe_CZ_5-1 and RtAbe_CZ_2-1, is more resistant than Ei-2 and Cvi-0, which contain only the resistance allele of the common QTL on chromosome 2. It may be envisaged that if RtAbe_CZ_2-1 and RtAbe_EZ_2-1 are allelic variants, then the presence of the stronger Ei-2 allele of this QTL in CIBC-5 may provide complete resistance.

A GO enrichment analysis of the genes within the physical interval of the RtAbe_CZ_5-1 QTL showed that biological process terms like defense response and response to stress were enriched significantly. A total of 53 defense-related genes were present in this interval that included a cluster of R genes. These R genes form a part of the major recognition gene complex MRC-J (Holub et al., 1997), which is involved in disease resistance. Some of the R genes in this region that are implicated in disease resistance against various pathogens include FLS2 (Zipfel et al., 2004; Sun et al., 2006), *RPW11* (Wilson et al., 2001), *RRS1* (Deslandes et al., 1998), *RPP8* (McDowell et al., 1998), and *RPS4* (Hinsch and Staskawicz, 1996). The presence of multiple R genes in this interval may indicate a possibility of effector-triggered immunity (ETI) or effector-triggered susceptibility (ETS) in *Arabidopsis* for *A. brassicae*. However, the role of ETI in plant-necrotrophic interactions is not well-documented except RLM3, which triggers ETI against *Leptosphaeria maculans* (Staal et al., 2008). The effectors of various necrotrophic pathogens activate certain signaling cascades to cause hypersensitive (HR) cell death, thus leading to susceptibility (ETS) (Lorang et al., 2004, 2007).

Interestingly, susceptibility/resistance to other necrotrophs has been mapped to the physical intervals similar to that of the RtAbe_CZ_5-1 QTL. In the case of the necrotrophic pathogen, *Botrytis cinerea*, one of the QTLs identified for susceptibility falls into the same physical region (Denby et al., 2004). Similarly, QRP3, a QTL governing resistance to *Plectosphaerella cucumerina*, a necrotrophic fungus, is located in the same region (Llorente et al., 2005). The co-localization of QTLs for different necrotrophic pathogens at this region indicates that there might be a common resistance/susceptibility factor that might be involved in basal defense against these necrotrophs.

Similarly, the region is also enriched for many plant defensins and defensin-like genes. Defensins are a group of antimicrobial peptides found across the plant species. They have repeatedly been shown to have antimicrobial activity *in vitro* against a spectrum of bacteria and fungi. A well- studied defensin, *PDF1.2*, which is activated by methyl jasmonate and ethylene, also lies in the QTL interval of RtAbe_CZ_5-1. *PDF1.2* is a marker of jasmonic acid and ethylene signaling pathways, and accumulation of *PDF1.2* has been implicated in resistance against various necrotrophs (Glazebrook, 2005; Spoel et al., 2007).

However, the list of candidate genes controlling resistance cannot be limited to genes with known functions in disease because resistance/susceptibility to the pathogen may involve any aspect of plant biology. Therefore, further fine mapping efforts are required to identify the gene(s) responsible for resistance. Additionally, the results reported here encourages the screening of a wider range of *Arabidopsis* accessions and employing a genome-wide association study design to gain a population-wide view of the genetic architecture of disease resistance.

## Conclusion

*Arabidopsis* accessions have considerable variation for resistance to *A. brassicae*, thus providing a good model to study the genetics of resistance. This is the first comprehensive study of quantitative phenotypic variation in response to *A. brassicae* leading to the identification of the loci governing resistance. QTL mapping in the three populations revealed multiple loci to be involved in resistance, thus confirming the quantitative nature of host responses to necrotrophic pathogens. The presence of different major QTLs in different populations leads us to hypothesize that the resistance to *A. brassicae* may be conferred by different genes/mechanisms that might be activated in various accessions of *Arabidopsis*. The overlapping QTL in all the three populations had different effect sizes in different populations, thus indicating that gene(s) in that locus would have varying effects on resistance depending on the genetic background. The study has also shed light on few facets of the genetic architecture of resistance to *A. brassicae viz*. effect size of the genetic factors identified and the additive nature of resistance. The identification of the gene(s) in the major QTLs and the mechanism underlying the resistance conferred by the gene(s) may, therefore, provide potential breeding targets for disease resistance against *Alternaria* blight in related *Brassica* species.

## Author Contributions

DP, AP, and JK designed the study. AK and VG developed and analyzed the data for the RIL population and performed the QTL analysis. SR developed the two F_2_ populations, analyzed the data and performed the QTL analysis for the F_2_ populations. DP, AP, JK, VG, SR all contributed to writing the manuscript. All the authors approved the final version of the manuscript.

## Conflict of Interest Statement

The authors declare that the research was conducted in the absence of any commercial or financial relationships that could be construed as a potential conflict of interest.

## Abbreviations

CDI: Cumulative Disease Index
DI: Disease Index
GO: gene ontology
LOD: Logarithm of Odds
NCDI: Normalized Cumulative Disease Index
NDI: Normalized Disease Index
QTL: quantitative trait locus
