# Supplementary material for: Genetic Architecture of Resistance to Alternaria brassicae in Arabidopsis thaliana: QTL Mapping Reveals Two Major Resistance-Conferring Loci
Source: Front Plant Sci. 2017 Feb 24;8:260. doi: 10.3389/fpls.2017.00260 (PMC5323384; doi:10.3389/fpls.2017.00260)
Supplement: Supplementary file 1 [file Table_1.DOCX]

**Supplementary Table 1:** List of 15 Arabidopsis accessions used in this study

| **Accession** | **Accession ID** | **Country of origin** |
| --- | --- | --- |
| CIBC-5 | 6908 | UK |
| Ei-2 | 6915 | Germany |
| Oy-0 | 6946 | Norway |
| Cvi-0 | 6911 | Cape Verde Islands |
| Wa-1 | 6978 | Poland |
| HR5 | 6924 | UK |
| Eden-2 | 6913 | Sweden |
| Mt-0 | 6939 | Libya |
| Kondara | 6929 | Tajikistan |
| Ll-0 | 6933 | Spain |
| Gre-0 | 7160 | USA |
| Zdr-1 | 6984 | Czech Republic |
| Kin-0 | 6926 | USA |
| Est-1 | 6916 | Russia |
| Ws-0 | 6980 | Russia |
