# Supplementary material for: Genetic Architecture of Resistance to Alternaria brassicae in Arabidopsis thaliana: QTL Mapping Reveals Two Major Resistance-Conferring Loci
Source: Front Plant Sci. 2017 Feb 24;8:260. doi: 10.3389/fpls.2017.00260 (PMC5323384; doi:10.3389/fpls.2017.00260)
Supplement: Supplementary file 2 [file Table_2.DOCX]

**Supplementary Table 2:** Summary of the genetic map generated using the CvG-RIL population

| **Chr** | **Total markers** | **Marker density/cM** | **Average interval size (cM)** | **Gaps (>10 cM)** | **Length (cM)** |
| --- | --- | --- | --- | --- | --- |
| 1 | 53 | 0.419968304 | 2.381132075 | 0 | 126.2 |
| 2 | 25 | 0.388198758 | 2.576 | 1 | 64.4 |
| 3 | 44 | 0.420007637 | 2.380909091 | 0 | 104.76 |
| 4 | 26 | 0.482374768 | 2.073076923 | 0 | 53.9 |
| 5 | 54 | 0.565445026 | 1.768518519 | 1 | 95.5 |
|  |  |  |  | **Total Length** | **444.76** |
