# Supplementary material for: Genetic Architecture of Resistance to Alternaria brassicae in Arabidopsis thaliana: QTL Mapping Reveals Two Major Resistance-Conferring Loci
Source: Front Plant Sci. 2017 Feb 24;8:260. doi: 10.3389/fpls.2017.00260 (PMC5323384; doi:10.3389/fpls.2017.00260)
Supplement: Supplementary file 3 [file Table_3.DOCX]

**Supplementary Table 3:** Summary of the genetic map generated using the CZF­_2_ population

| **Chr** | **Total markers** | **Marker density/cM** | **Average interval size (cM)** | **Gaps (>10 cM)** | **Length (cM)** |
| --- | --- | --- | --- | --- | --- |
| 1 | 33 | 0.336580142 | 3.06390625 | 1 | 98.045 |
| 2 | 28 | 0.445080273 | 2.419615385 | 0 | 62.91 |
| 3 | 27 | 0.309129628 | 3.49368 | 2 | 87.342 |
| 4 | 20 | 0.363517394 | 2.895684211 | 0 | 55.018 |
| 5 | 35 | 0.372300819 | 2.848787879 | 0 | 94.01 |
|  |  |  |  | **Total Length** | **397.325** |
