# Supplementary material for: Genetic Architecture of Resistance to Alternaria brassicae in Arabidopsis thaliana: QTL Mapping Reveals Two Major Resistance-Conferring Loci
Source: Front Plant Sci. 2017 Feb 24;8:260. doi: 10.3389/fpls.2017.00260 (PMC5323384; doi:10.3389/fpls.2017.00260)
Supplement: Supplementary file 4 [file Table_4.DOCX]

**Supplementary Table 4:** Summary of the genetic map generated using the EZF­_2_ population

| **Chr** | **Total markers** | **Marker density/cM** | **Average interval size (cM)** | **Gaps (>10 cM)** | **Length (cM)** |
| --- | --- | --- | --- | --- | --- |
| 1 | 33 | 0.336076259 | 2.975515152 | 0 | 98.192 |
| 2 | 21 | 0.319265386 | 3.132190476 | 0 | 65.776 |
| 3 | 20 | 0.27474037 | 3.6398 | 1 | 72.796 |
| 4 | 17 | 0.237808802 | 4.205058824 | 0 | 71.486 |
| 5 | 29 | 0.322735012 | 3.098517241 | 0 | 89.857 |
|  |  |  |  | **Total Length** | **398.107** |
