# Supplementary material for: Genetic Architecture of Resistance to Alternaria brassicae in Arabidopsis thaliana: QTL Mapping Reveals Two Major Resistance-Conferring Loci
Source: Front Plant Sci. 2017 Feb 24;8:260. doi: 10.3389/fpls.2017.00260 (PMC5323384; doi:10.3389/fpls.2017.00260)
Supplement: Supplementary file 5 [file Table_5.DOCX]

**Supplementary Table 5:** Summary of GO term enrichment analysis of genes in the RtAbe_CZ_5-1 QTL interval

| **GO term** | **Description** | **P-value^a^** | **FDR q-value^b^** | **Enrichment^c^** |
| --- | --- | --- | --- | --- |
| GO:0006952 | defense response | 2.01^-12^ | 6.50^-09^ | 3.47 |
| GO:0006950 | response to stress | 3.44^-08^ | 5.56^-05^ | 2.21 |

^a^ significance value of the term enrichment.

^b^ FDR (False discovery rate) represents multiple hypothesis corrected p-value.

^c^ Enrichment of the GO term with respect to the other terms.
